# Association of Facebook Use With the Physical and Mental Health of Undergraduate Students in Jalalabad City, Afghanistan: A Cross-Sectional Study

**DOI:** 10.7759/cureus.84922

**Published:** 2025-05-27

**Authors:** Abdul G Sherzad, Kamran Zafarzai, Mohammad Q Nabizada, Abdul S Hussaini, Abdul N Jabarkhil, Habibullah Mawlavyzada, Said N Saidy, Najeebullah Ameen, Salahuddin Rahimee, Shukrullah Sahak

**Affiliations:** 1 Department of Respiratory and Rheumatic Heart Diseases, Faculty of Medicine, Nangarhar University, Jalalabad, AFG; 2 Department of Internal Medicine, Nangarhar Regional Hospital, Jalalabad, AFG; 3 Division of Research, Compilation, and Translation, Spinghar Institute of Higher Education (Nangarhar), Jalalabad, AFG; 4 Department of Physiology, Faculty of Medicine, Nangarhar University, Jalalabad, AFG; 5 Department of Cardiorespiratory Diseases, Faculty of Medicine, Kabul University, Kabul, AFG; 6 Department of Internal Medicine, Aliabad Teaching Hospital, Kabul, AFG; 7 Department of Tuberculosis and Infectious Diseases, Faculty of Medicine, Nangarhar University, Jalalabad, AFG; 8 Department of Pediatrics, Faculty of Medicine, Nangarhar University, Jalalabad, AFG

**Keywords:** adverse health affects, facebook, mental health, physical health, undergraduate students

## Abstract

Background/objectives

Facebook is considered one of the most widely used social media platforms among young adults worldwide, including undergraduate students. Addiction to Facebook has effects on users’ physical and mental health. Therefore, the primary aim of this study is to investigate the association between the frequency of Facebook use and its impact on the physical and mental health of undergraduate students in Jalalabad City, Afghanistan.

Methods

A multi-institutional cross-sectional study was conducted on 489 students from public and private universities from May 2024 to December 2024. A non-probability convenience sampling technique was used to recruit study participants.

Results

A total of 489 participants were enrolled in this study. 406 (83%) were males and 83 (17%) were females. More than 371 (70%) of them use Facebook for up to two hours a day. Students who spent up to two hours on Facebook were more likely to experience various physical and mental health issues, such as become angry when Facebook use is interrupted (p = 0.001), experience eye pain (p = 0.022), continuing to use Facebook despite having an urge for urination (p = 0.001), feeling of loneliness (p = 0.008), insomnia (p = 0.038), getting irritated with family members when they say something while using Facebook (p = 0.001), and postponing meals (p = 0.001). Moreover, gender (p = 0.024) and faculty of the students (p = 0.002) had a significant relationship with the duration of Facebook use.

Conclusions

The study concluded that excessive Facebook use among students has negative effects on their physical and mental health. Moreover, individuals who use Facebook more frequently tend to be more addicted to it than others. Therefore, higher education institutions need to implement health promotions and interventions to support youth in the healthy use of Facebook, and screening for eye and musculoskeletal conditions is advised.

## Introduction

Facebook is one of the social websites where people from different countries log in and connect with each other. The number of users of social networking sites (SNS) is growing rapidly. There are numerous social networks, such as Facebook, WhatsApp, YouTube, Instagram, and so on; the most widespread among them is Facebook [1]. Facebook was launched in 2004 by Mark Zuckerberg and his friends and was initially only used by Harvard University students. But Facebook has evolved into a social network over time [2]. Facebook with 3,070 million monthly active users (MAUs), YouTube with 2,530 million, Instagram with 2,000 billion, WhatsApp with 2,000 million, TikTok with 1,590 million, WeChat with 1,380 million, Telegram with 950 million, Facebook Messenger with 947 million, Snapchat with 850 million, Douyin with 766 million, and Kuai Shou with 714 million are the most popular social media accessed in the world as of February 2025 [3].

According to a report published in January 2024, Afghanistan is home to 42.80 million people, of whom 7.88 million use the internet. In 2024, 18.4% of Afghanistan's population had access to the internet. According to Meta advertising resources, Facebook had 4.05 million users in Afghanistan at the beginning of 2025. As of early 2025, 83% of Facebook's Afghan advertising audience was male and 17% female [4]. A 2023 survey in the United States found that 83% of adults use YouTube, 69% use Facebook, and 25% use Snapchat [5]. In 2022, users worldwide spent an average of 19.42 hours per month using the Facebook mobile application [6]. A study on Facebook addiction and its relationship to mental health among students in Thailand found that Facebook addiction is associated with abnormal mental health status, somatic symptoms, anxiety, insomnia, and social problems, which may be associated with dysfunction and severe depression [7]. Similarly, research conducted in Bangladesh found that more than half of the students used Facebook for more than two hours daily, leading to various physical and mental health problems. Students became angry when interrupted while using Facebook; headaches, eye pain, blurred eyes, insomnia, and difficulty waking up may also occur. Additionally, the majority of them said they occasionally used Facebook during mealtime, and they became excited when they saw something unexpected on Facebook [2,8].

In addition to these findings, a study conducted by previous authors also reported that excessive Facebook use is associated with behavioral problems and poor academic performance in university students, affecting their lifestyle and interactions with others. In addition, Facebook addiction is directly correlated with anxiety and depression among university students, affecting their mental health and social life. [9,10]. Furthermore, a study has examined the negative effects of using Facebook among university students. According to the results, 39% of respondents believed that using Facebook had a negative impact on their academic or professional performance. The study also claimed that 37% of respondents said their prolonged use of Facebook prevented them from learning new knowledge or skills [11]. Excessive use of Facebook also leads to deterioration in physical and mental health. It affects their lifestyle, thoughts, beliefs, and norms and leads to loneliness, lack of self-confidence, and insomnia. Similarly, the use of social media worsens mental and physical health. It affects how they live, think, believe, and behave, making them lonely, insecure, and prone to insomnia.

Unfortunately, young adults use social media more often than anyone else and are at disproportionate risk for mental health problems [7]. Currently, university students' academic performance can be significantly affected by excessive social media use. Therefore, understanding the extent of Facebook use and highlighting its connection to physical and mental health is an extremely powerful way to suggest approaches to address this problem. However, there are no relevant studies on the association of Facebook use with the physical and mental health of university students in Jalalabad city, Afghanistan. Thus, the primary aim of this study is to investigate the association between the frequency of Facebook use and its impact on the physical and mental health of undergraduate students in Jalalabad City, Afghanistan.

## Materials and methods

Study setting and design

This cross-sectional survey was carried out between May 2024 and December 2024 among 489 male and female students at public and private universities in Jalalabad city, Afghanistan. The following criteria were used to determine who was eligible and ineligible to participate: 1) students from all academic years; 2) students from public and private universities in Jalalabad city, Afghanistan, who were registered with the Ministry of Higher Education; 3) students who were willing to participate in the study; 4) having internet access and being at least 18 years of age; 5) using Facebook for at least 35 minutes per day were included. 1) Students undergoing counseling or psychological treatment; 2) incomplete questionnaires; and 3) individuals who do not complete the questionnaire are automatically excluded. 

Sample size and sampling technique

The study used Cochran’s formula for an infinite population. The sample size for the non-probability convenience sample was determined using the formula $\left( n_{0}=\frac{z^{2}pq}{e^{2}} \right)$, where n represents the sample size, z is the standard normal z-score, which corresponds to a 95% confidence interval (CI) of 1.96, p is estimated proportion of an attribute that is present in the population, which is taken as 50% [8], Q= 1-P (1-0.5), and e^2^ is the margin of error (desired level of precision) set at 5% (0.05). This formula was used to determine a minimum sample size of 384 participants.

Calculation: $n_=\frac{z^{2}pq}{e^{2}}=\frac{\left( 1.96 \right)^2\left( 0.5\ast 0.5 \right){}}{\left( 0.05 \right)^2{}}=384$

In order to boost the study's statistical power and account for the possible statistical non-response rate, the minimum required sample size of 384 was raised by 15%.

Adjusted sample size $= 384 + (15\% \text{ of } 384) = 384 + 57.6 \approx 384 + 58 = 442$

The extra 15% amounts to 57.6, which was rounded to the nearest whole number. Thus, a total of 384 + 58 = 442 participants were needed to be nominated for the study. Ultimately, 489 students completed the questionnaire during the study period.

Data collection and patient characteristics

The questionnaire was taken with prior permission from a previous study by Rahman and Zakaria, 2021 [8]. To achieve our study's objectives, the questionnaire was translated into Pashto, the country's official language, and the context was slightly adapted. Before the questionnaires were distributed, the respondents were informed about the objectives of the study. The questionnaire link was distributed online through various social media platforms such as email, Facebook pages, Telegram accounts, Instagram, WhatsApp, and other students’ association networks. Social media groups and student organizations were targeted to ensure diverse participation and equal representation across universities. The questionnaire was divided into three sections: questions about respondents' sociodemographic characteristics; questions about purpose and usage behavior; and questions about the impact of Facebook on their physical and mental health.

Measurement

Questions validated in the context of Afghanistan were asked to assess the health effects of Facebook; these were determined using 14 questions on health effects, with five-point Likert scales: strongly agree, agree, neutral, disagree, and strongly disagree. For the chi-square test, the scale was recoded with three categories: agree, neutral, and disagree.

Pilot study

The questionnaire was pre-tested on a small sample of 50 respondents to confirm its validity and reliability. The Cronbach's alpha test was used to assess the reliability of the tool. The results demonstrated the recognized reliability of the tool: 0.79 for questions about purpose and usage behavior and 0.83 for questions about the effects of Facebook use on their physical and mental health. The pilot study observation was excluded from the final analysis.

Statistical analysis

Data were analyzed using SPSS version 28.0 (IBM Corp., Armonk, USA). Quantitative data were summarized using means ± standard deviations or medians and interquartile ranges (IQRs) for continuous variables, depending on distribution. All qualitative information was documented as percentages and frequencies (n) of responses. Additionally, chi-square analysis (χ2) was used to assess the association between the independent and primary outcome variables.

## Results

Demographic characteristics of study participants

A total of 489 respondents were enrolled, comprising 406 (83%) male and 83 (17%) female students. The mean age of the participants was 22 ± 2.7 years (range, 18-38 years). Of them, 373 (76.3%) attended public universities, 116 (23.7%) attended private universities, and 318 (65%) were medical faculty members at both public and private universities. Only 20 (4.1%) of respondents said they watched TV regularly, compared to 32 (6.5%) who said they read the newspaper regularly. On average, 371 (75.9%) of students used Facebook for up to two hours a day, and 118 (24.1%) used it for more than two hours. While 366 (74.8%) also agreed that they became excited when they saw something unexpected, such as a sudden announcement, a spontaneous live video, a sudden viral post, or an unexpected comment from someone they hadn't heard from in a long time on Facebook. Table 1 shows the statistics of the basic information of the study participants.

**Table 1:** Demographic characteristics of study participants (N = 489)

Variables	Frequency	Percentage
Gender		
Male	406	83
Female	83	17
Age Category (by years)		
18-30	480	98.2
31-40	7	1.4
> 40	2	0.4
Marital Status		
Married	87	17.8
Unmarried	402	82.2
Place of residence of the students		
Public Hostel	117	23.9
Private Hostel	132	27%
One's residence	240	49.1
Area of residence of the family		
Urban	302	61.8
Rural	187	38.2
University Type		
Public	373	76.3
Private	116	23.7
Faculty of the respondents		
Practical	337	68.9
Theoretical	152	31.1
Income		
High	16	3.3
Medium	390	79.8
Low	83	17
Reading newspaper		
Irregular	139	28.4
Sometimes	318	65
Regular	32	6.5
TV Viewing		
Irregular	119	24.3
Sometimes	350	71.6
Regular	20	4.1
Students average Facebook using time		
Up to 2 hours	371	75.9
> 2 hours	118	24.1
Students father education		
Illiterate	82	16.8
Primary	55	11.2
Secondary	42	8.6
Higher School	103	21.1
University	133	27.2
Master	74	74
Students mother education		
Illiterate	186	38
Primary	55	11.2
Secondary	25	5.1
Higher School	41	8.4
University	172	35.2
Master	10	2

Students’ health effects of Facebook use

**Figure 1:**
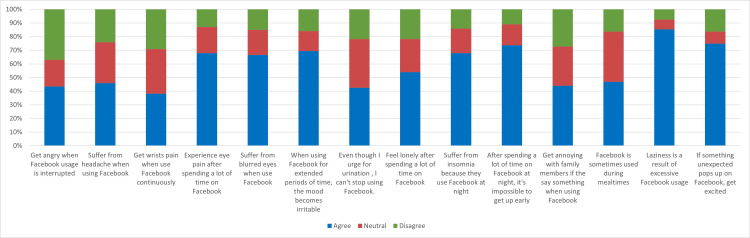
Students' health effects of Facebook use (N = 489)

Figure 1 shows that 418 (85.5%) of the respondents reported that excessive Facebook use caused laziness in their lives. In addition, 360 (73%) respondents revealed that they cannot wake up early in the morning when using Facebook for a long time at night, while 366 (74 %) also agreed that they became excited when they see something unexpected on Facebook. Of the respondents, 340 (69.5%) stated that their mood became irritable when they used Facebook continuously, while 334 (68%) respondents reported that they were suffering from blurred eyes by using Facebook excessively. Most of the participants 332 (67.9%) reported that they were having eye pain when using Facebook for a long time. Furthermore, 323 (67.9%) participants acknowledged that they had insomnia as they were connected with Facebook for a long time late at night.

Distribution of the health effects of Facebook use by the duration of use

According to the study's results, students who used Facebook for more than two hours a day were significantly more likely to experience adverse health effects. The items included becoming angry when their using Facebook is interrupted (χ2 = 25.305; p = 0.001), eye pain after prolonged Facebook use (χ2 = 7.594; p = 0.022), continuing Facebook use despite having the urge for urination (χ2 = 19.830; p = 0.001), feeling lonely (χ2 = 9.762; p = 0.008), suffering from insomnia (χ2 = 6.551; p = 0.038), getting annoyed with family members if they say something when using Facebook (χ2 = 28.958; p = 0.001), and Facebook is sometimes used during mealtimes (χ2 = 26.171; p = 0.001) (Table 2).

**Table 2:** Students' health effects by the duration of Facebook use (N= 489)

Variables	Duration of Facebook Use					
	Up to 2 Hours n (%)	>2 Hours n (%)	χ^2^			P
Get angry when Facebook usage is interrupted						
Agree	138 (37.2)	212 (43.4)	25.305			0.001
Neutral	85 (22.9)	96 (19.6)				
Disagree	148 (39.9)	181 (37)				
Suffer from headaches when using Facebook						
Agree	166 (44.7)	225 (46)	5.867			0.053
Neutral	121 (32.6)	146 (29.9)				
Disagree	84 (22.6)	118 (24.1)				
Get wrist pain when using Facebook continuously						
Agree	132 (35.6)	187 (38.2)	4.750		0.093	
Neutral	128 (34.5)	160 (32.7)				
Disagree	111 (29.9)	142 (29)				
Experience eye pain after spending a lot of time on Facebook						
Agree	240 (64.7)	332 (67.9)	7.594		0.022	
Neutral	80 (21.3)	94 (19.2)				
Disagree	51 (13.7)	63 (12.9)				
Suffer from blurred eyes when using Facebook						
Agree	247 (66.6)	334 (68.3)	3.396		0.183	
Neutral	71 (19.1)	85 (17.4)				
Disagree	53 (14.3)	70 (14.3)				
When using Facebook for extended periods of time, the mood becomes irritable						
Agree	252 (67.9)	340 (69.5)	1.943		0.379	
Neutral	56 (15.1)	71 (14.5)				
Disagree	63 (17)	78 (16)				
Even though I urge for urination, I can't stop using Facebook						
Agree	137 (36.8)	208 (42.5)	19.830		.001	
Neutral	145 (39.1)	175 (35.8)				
Disagree	89 (24)	106 (21.7)				
Feel lonely after spending a lot of time on Facebook						
Agree	187 (50.4)	264 (54)	9.762		0.008	
Neutral	93 (12.1)	119 (24.3)				
Disagree	91 (24.5)	106 (21.7)				
Suffer from insomnia because they use Facebook at night						
Agree	242 (65.2)	332 (67.9)	6.551		0.038	
Neutral	69 (18.6)	88 (18)				
Disagree	60 (16.2)	69 (14.1)				
After spending a lot of time on Facebook at night, it's impossible to get up early						
Agree	265 (71.4)	360 (73.6)	3.869		0.144	
Neutral	61 (16.4)	75(15.3)				
Disagree	45 (12.1)	54 (11)				
Get annoyed with family members when they say something while using Facebook						
Agree	138 (37.2)	215 (44)	28.958		.001	
Neutral	122 (32.9)	141 (28.8)				
Disagree	111 (29.9)	133 (27.2)				
Facebook is sometimes used during mealtimes						
Agree	150 (40.4)	229 (46.8)	26.171	.001		
Neutral	156 (42)	180 (36.8)				
Disagree	65 (17.5)	80 (16.4)				
Excessive use of Facebook creates laziness						
Agree	314 (84.6)	418 (85.5)	0.910	0.634		
Neutral	27 (7.3)	34 (7)				
Disagree	30 (8.1)	37 (7.6)				
If something unexpected pops up on Facebook, get excited						
Agree	273 (73.6)	366 (74.8)	1.891	0.388		
Neutral	36 (9.7)	43 (8.8)				
Disagree	62 (16.7)	80 (16.4)				

Distribution of sociodemographic characteristics by duration of Facebook use

Table 3 shows a significant association with the duration of Facebook use and the students' faculty (χ2 = 9.253; p = 0.002) and gender (χ2 = 5.109; p = 0.024). However, this study found no association between the duration of Facebook use and other sociodemographic variables.

**Table 3:** Distribution of sociodemographic characteristics by the duration of Facebook use (N = 489)

Variables	Duration of Facebook Use			
	Upto 2 Hours n (%)	>2 Hours n (%)	χ^2 ^	P
Gender				
Male	300 (80.9)	106 (89.8)	5.109	0.024
Female	71 (19.1)	12 (10.2)		
Marital status				
Married	64 (80.5)	23 (19.5)	0.307	0.579
Unmarried	307 (82.7)	95 (17.3)		
Place of residence of the student				
Public Hostel	90 (24.3)	27 (22.9)	0.564	0.754
Private Hostel	97 (26.1)	35 (26.5)		
One's residence	184 (49.6)	56 (23.3)		
Area of residence of the family				
Urban	235 (63.3)	67 (56.8)	1.633	0.201
Rural	136 (36.7)	51 (43.2)		
University type				
Public	288 (77.6)	85 (72)	1.548	0.213
Private	83 (22.4)	33 (28.4)		
Faculty of the respondents				
Practical	269 (72.5)	68 (57.6)	9.253	0.002
Theoretical	102 (27.5)	50 (42.4)		
Income				
High	9 (2.4)	7 (5.9)	5.110	0.078
Medium	303 (81.7)	87 (73.7)		
Low	59 (15.9)	24 (20.3)		
Reading newspaper				
Irregular	97 (26.1)	42 (35.6)	5.561	0.062
Sometimes	246 (66.3)	72 (61)		
Regular	28 (7.5)	4 (3.4)		
TV viewing				
Irregular	86 (23.2)	33 (28)	1.161	0.560
Sometimes	270 (72.8)	80 (67.8)		
Regular	15 (4)	5 (4.2)		
Student's father's education				
Illiterate	57 (15.4)	25 (21.2)	5.185	0.394
Primary	38 (10.2)	17 (14.4)		
Secondary	31 (8.4)	11 (9.3)		
Higher School	83 (22.4)	20 (19.4)		
University	104 (28)	29 (21.8)		
Master's	58 (15.6)	16 (21.6)		
Students mother education				
Illiterate	147 (39.6)	39 (33.1)	4.809	0.440
Primary	40 (10.8)	15 (12.7)		
Secondary	21 (5.7)	4 (3.4)		
Higher School	30 (8.1)	11 (9.3)		
University	124 (33.4)	48 (40.7)		
Master's	9 (2.4)	1 (0.8)		

## Discussion

This study investigated the frequency of Facebook use among students at public and private universities, as well as its effects on their physical and mental health. 

A total of 489 participants were enrolled in the study. The majority of participants were male. In contrast to the findings of previous studies, most students in this study reported using Facebook for an average of two hours per day [2,8,10,11]. This discrepancy can be explained by several factors as follows: students in our study may prioritize academic responsibilities or daily tasks and limit their time on social media. Limited internet access, high data costs, or inconsistent electricity supply could also play a role. Additionally, the timing of our study, possibly during periods of high academic activity such as exams or assignments, may have influenced usage behavior. Many students may use Facebook specifically for academic or communication purposes, resulting in shorter sessions, while others may prefer alternative platforms such as Instagram or TikTok. These combined factors provide a realistic explanation for the average Facebook usage of two hours per day observed in our study. Similar findings were observed in study conducted among students at Dow University in Pakistan [12 ]. 

Regarding residence, the majority of them lived at home compared to public and private dormitories. A study among students from King Abdulaziz University, Saudi Arabia, found similar results [13]. Furthermore, most of the respondents' families were from the urban area compared to the rural area. This result contradicts the result of a previously published study [8,14]. The difference can be attributed to several factors. First, our study was conducted in institutions primarily accessible to urban students, such as city-based universities, which naturally attract more participants from urban areas. Additionally, urban regions often provide better access to educational institutions and resources, leading to a higher concentration of students from these areas. These regional differences help explain the greater digital engagement and lower levels of traditional media consumption (such as newspapers and television) observed in our findings.

The present study also revealed that only a minority of respondents regularly read newspapers, whereas some watched TV regularly. This result aligns with earlier research carried out in Bangladesh [8]. A large proportion of participants reported experiencing health-related issues associated with excessive Facebook use, including laziness, irritability, eye-related problems such as blurred vision and eye pain, as well as insomnia, headaches, and wrist pain. Nearly half of the respondents (specifically, 46.8%) reported using Facebook during mealtimes, while the other stated that they became angry when their use of Facebook was interrupted by family members. These findings are in line with a study by Rahman and Zakaria, which found that over 50% of students spend more than two hours a day on Facebook, which can lead to a number of health problems. For example, students may become angry when their usage is interrupted; they may experience headaches, eye pain, blurred vision, or insomnia; when they come across something unexpected on Facebook, they may be excited; and most of them may report that meal times occasionally pass while they are on Facebook [8].

Previous studies have reported that Facebook can cause negative effects such as headaches, back pain, weight changes, and eye problems. Consistent with our findings, increased Facebook usage can lead to eye irritation, musculoskeletal pain, and missed meals [15, 16]. There is evidence from previous research to support our findings that students with urban-based parents, higher educational qualifications, and those who read newspapers irregularly are more likely to use Facebook over a longer period of time [8]. In addition, the results of this study also showed that the young population is more likely to engage in addictive Facebook behavior. Consistent findings were also reported in studies conducted in Pakistan and Nepal [10,15]. The prevalence rate of wrist pain reported in the current study was 38.2%, lower than the prevalence rates of 66% and 61% reported by students in Malaysia and Ghana, respectively [17,18].

Although it is difficult to explain some of the differences between the high prevalence of musculoskeletal pain reported in this study and other studies, cultural patterns in which students from different countries talk about musculoskeletal problems can be an explanation. In some countries, students may be more likely to complain, while in others, they may downplay the frequency. The current study found that medical students were more likely to use Facebook in a problematic way compared to other university students, which has not been examined in any previous studies. Facebook is perhaps the most important source of relief for medical students in their rigorous coursework, which is typically more demanding than that of other students [14,19]. A significant number of participants reported experiencing health-related issues associated with Facebook use, including laziness, irritability, eye-related problems such as blurred vision and eye pain, as well as insomnia, headaches, and wrist pain. Nearly half of the respondents also reported using Facebook during mealtimes, and others said they became angry when their use of Facebook was interrupted by family members. These findings are consistent with the results of previously published studies [5,8,20-22].

Furthermore, certain health-related items were found to be significantly associated with students' two-hour daily Facebook use, while other items had no influence. This disparity might result from variations in the sociodemographic characteristics and other behavioral elements that affect the study participants. The results of this study are consistent with those of other previously published studies [8,18].

Strengths and limitations of the study

To the best of our knowledge, this is the first study conducted in Jalalabad City addressing the association between Facebook use and the physical and mental health of undergraduate students, thereby filling a significant gap in local research. Second, it contributes to the existing literature by addressing a knowledge gap related to the impact of social media on student health in this specific context. Third, the study clearly demonstrates the extent of Facebook's impact on both the mental and physical health of students. Fourth, this study also establishes a significant relationship between Facebook use and students’ physical and mental health. Finally, the findings can assist policymakers in designing and implementing effective strategies. Specifically, policymakers can use the results of this research to develop targeted interventions aimed at promoting the responsible use of social media, particularly Facebook, and mitigating its negative effects on the physical and psychological well-being of youth.

There were several limitations to this study. First, we could not establish a causal relationship between the variables because the study was cross-sectional in nature. Future research employing analytical designs could provide more vigorous evidence of the causal relationships. Another major limitation is that we primarily attempted to determine the effects of Facebook use on mental and physical health using a limited number of variables, which may not have been sufficient to yield fully accurate results. Additionally, social desirability bias and other respondent-related factors may have skewed the responses. Furthermore, the study sample consisted exclusively of undergraduate students from universities in Jalalabad City, Afghanistan. Therefore, university students in other parts of Afghanistan may not be represented in our findings. It is also important to note that these results likely apply only to undergraduate students and may not accurately reflect the association between Facebook use and unhealthy habits in other age groups or among non-university populations of the same age. Finally, several selection biases may have arisen, as the study was conducted online, employed convenience sampling, and had a relatively small sample size. These factors may limit the generalizability of the results. A prospective study with a large sample size is needed to validate the association of Facebook use with the physical and mental health of undergraduate students.

Recommendations

First, universities should implement specific public awareness programs to make students aware of the potential harm caused by the use of social networks, specifically Facebook. Second, students should be encouraged to set specific limits for social networking, specifically Facebook, and to prioritize academic studies and personal health through apps that monitor screen time. Third, universities should organize dedicated workshops on the responsible use of social networks to help students understand how to use them in a way that does not cause harm to their mental and physical health. Fourth, students should be encouraged to take part in physical, social, and entertaining activities that can help reduce excessive use of Facebook and improve overall well-being. Fifth, universities should strengthen counseling and mental health services to help students cope with psychological problems such as anxiety, stress, and other mental effects linked to Facebook use.

Sixth, encourage healthy social media behaviors through public health campaigns highlighting the importance of balanced online and offline activities. Seventh, in order to ensure the mental health of students, parents and university authorities should work together to encourage them to maintain a balanced relationship with Facebook and with real life, and to adopt clear policies on the use of Facebook. Eighth, encourage university students to learn digital literacy to use social media, specifically Facebook, in a responsible and safe manner, limiting their exposure to hate speech and explicit content. Ninth, in order to improve academic performance, support remote learning so that students can balance their academic and entertaining activities on digital platforms. Additionally, further research should be conducted to examine the long-term effects of social network addiction and develop strategies for effective intervention so students can use social media in a healthy way.

## Conclusions

The study concluded that excessive Facebook use among students has negative effects on their physical and mental health. Most students experience various problems, such as eye pain, headaches, blurred vision, feeling alone, insomnia, difficulty waking up early in the morning, and excitement when they see something unexpected on Facebook. According to this study, individuals who use Facebook more frequently tend to be more addicted to it than others, which affects their physical and mental health in various ways. This study suggests that increasing student awareness of the connection between health and social media like Facebook should be prioritized, and when using it, safety measures should be taken into account. Therefore, higher education institutions need to implement health promotions and interventions to support youth in the healthy use of Facebook, and screening for eye and musculoskeletal conditions is advised.
